# Identification of Epithelial-Mesenchymal Transition-Related lncRNAs that Associated With the Prognosis and Immune Microenvironment in Colorectal Cancer

**DOI:** 10.3389/fmolb.2021.633951

**Published:** 2021-04-01

**Authors:** Chuan Liu, Chuan Hu, Jianyi Li, Liqing Jiang, Chengliang Zhao

**Affiliations:** ^1^ Graduate College, China Medical University, Shenyang, China; ^2^ Department of Spinal Surgery, The Affiliated Hospital of Qingdao University, Qingdao, China

**Keywords:** epithelial-mesenchymal transition, lncRNA, colorectal cancer, immune cells, prognosis

## Abstract

**Background:** The expression of long non-coding RNA (lncRNA) is associated with the epithelial-mesenchymal transition (EMT) in tumorigenicity, but the role of EMT-related lncRNA in colorectal cancer (CRC) remains unclear.

**Methods:** The clinical data and gene expression profile of CRC patients were obtained from The Cancer Genome Atlas database. Differential expression analysis, Cox regression model, and Kaplan-Meier analysis were used to study the relationship between EMT-related lncRNAs and the prognosis of CRC. Functional analysis and unsupervised clustering analysis were performed to explore the influence of certain lncRNAs on CRC. Finally, Cytoscape was used to construct mRNA-lncRNA networks.

**Results:** Two signatures incorporating six and ten EMT-related lncRNAs were constructed for predicting the overall survival (OS) and disease-free survival (DFS), respectively. Kaplan-Meier survival curves indicated that patients in the high-risk group had a poorer prognosis than those in the low-risk group. The results of the functional analysis suggested that the P53 and ECM-receptor pathways affect the prognosis of CRC, and AL591178.1 is a key prognostic EMT-related lncRNA, which is negatively related to immune cells, P53 pathway, and ECM-receptor pathway.

**Conclusion:** Six OS-related and ten DFS-related EMT-related lncRNAs were correlated with the prognosis of CRC by potentially affecting the immune microenvironment, and AL591178.1 plays a key role as a prognostic factor.

## Introduction

Epithelial-mesenchymal transition (EMT) is a dynamic and reversible process that occurs in embryogenesis, organ development, wound healing, and fibrosis (Hay, 2005; Kalluri and Weinberg, 2009; Zeisberg and Neilson, 2009). It is characterized by the interaction between polarized epithelial cells and the basement membrane. Different processes make them show different mesenchymal cell phenotypes, including invasiveness migration, anti-apoptosis, and high levels of extracellular matrix (ECM) (Kalluri and Neilson, 2003). More importantly, there is evidence that the dysregulation of EMT plays an important role in the occurrence and progression of cancers, especially in the process of invasion, metastasis, and resistance of antineoplastic drug (Montemayor-Garcia et al., 2013; Zhao et al., 2013; Nieto et al., 2016). Recent studies have shown that the intermediate state of EMT is partial EMT (p-EMT), which is involved in the tumor progression and promotes the migration of tumor cells and the formation of circulating tumor cell clusters (Aiello et al., 2018; Saitoh, 2018). Besides, the process of EMT is regulated by various genes to affect the tumor progression (Huang et al., 2020; Xie et al., 2020). Thus, it is essential to further explore the function of the EMT-related genes in cancers.

With the emergence of next-generation sequencing technology, comprehensive genomic atlas based on tumor molecular characteristics have been received extensive research (Sanchez-Vega et al., 2018; Nacev et al., 2019). Different genomics, including mRNA, microRNA, long non-coding RNA (lncRNA), and other molecules, help to broaden our understanding of genomic characteristics in different tumors. A series of studies have proved that lncRNAs play an important role in the progression of tumors and can be used as robust predictors of the prognosis for cancer patients (Wu et al., 2020; Zhuang et al., 2020). Interestingly, the EMT mechanism of lncRNA is involved. For example, lncRNA ADAMTS9-AS1 is an EMT-related gene and is associated with lymph node invasion and prognosis of colorectal cancer (CRC) (Chen W. et al., 2020). Additionally, increased expression of lncRNA PTAR can promote EMT and metastasis in tumorigenicity of ovarian cancer cells (Liang et al., 2018). However, most research mainly focused on the impact of a single lncRNA on tumors, and it is necessary to conduct a comprehensive analysis of the potential role of EMT-related lncRNAs on cancer prognosis.

CRC is the most common gastrointestinal adenocarcinoma, with 1.8 million new cases occurring every year and causing approximately 900,000 deaths (Bray et al., 2018). In recent years, morbidity and mortality have been on the rise in many countries, especially in Asian (Chen et al., 2016). Although the improvement of early diagnosis, immunotherapy, and chemotherapy has significantly improved the diagnosis and treatment of CRC, the survival rate is still not ideal (Cunningham et al., 2010). Due to the molecular heterogeneity of CRC cells, it is common to get completely different clinical outcomes (Guinney et al., 2015). Therefore, it is imperative to explore the molecular mechanisms of the prognosis for CRC patients. In particular, EMT is a field associated with lncRNA expression and cancer development, which may lead to a deeper understanding of this kind of tumor and provide new insights.

## Methods

### Data Collection and Processing

We obtained the gene expression profile of CRC patients from the TCGA database (https://cancergenome.nih.gov/), and the corresponding clinical information were obtained from the cBioportal database (https://www.cbioportal.org/) (Cerami et al., 2012). According to the gene annotation in the GENCODE project (https://www.gencodegenes.org/) (Derrien et al., 2012), a list of lncRNA was distinguished. Then the clinical data of CRC patients were processed for further analysis, including age, gender, tumor grade, AJCC TNM stage, follow-up time and follow up data. All these data are publicly available, thus no specific ethical approval and informed consent are required and the work is exempt.

### Identification of Differentially Expressed Epithelial-Mesenchymal Transition-Related lncRNA

The list of EMT-related genes (ERGs) was downloaded from the EMT gene database (http://dbemt.bioinfo-minzhao.org/download.cgi). Pearson correlation analysis was used to identify EMT-related lncRNAs (correlation coefficient |r| > 0.4 and *p* < 0.05). Then, differentially expressed analysis was performed by comparing the lncRNA expression of tumor samples with normal samples using the “limma” package in the R software (Ritchie et al., 2015), differentially expressed EMT-related lncRNAs would be confirmed when FDR < 0.05 and |logFC| > 1.

### Construction and Validation of Prognostic Signatures

In order to identify differentially expressed EMT-related lncRNAs associated with the overall survival (OS) and disease-free survival (DFS), 533 CRC patients with both OS and DFS information were included. Firstly, all CRC patients were randomly divided into a training set (70%) and a validation set (30%). The training set was used to perform survival analyses and establish prognostic signatures, while training set was used to validate corresponding signature. The univariate Cox analysis was then performed to select OS- and DFS-related differentially expressed EMT-related lncRNAs, and lncRNAs with *p* value < 0.05 were listed as candidate prognostic factors. Next, multivariate Cox analysis was used to select the most appropriate OS-related or DFS-related differentially expressed EMT-related lncRNAs and construct two signatures. The corresponding risk scores of each CRC patients in both training and validation sets were calculated simultaneously. The formula is as follows:

$$Risk\;Score\;=\;\sum_{i=0}^{n}\beta_{i}{\text{ * G}}_{i}$$

Here, ‘$\beta_{i}$’ is the estimated regression coefficient of the gene from the multivariate Cox proportional hazards regression analysis, and ‘$G_{i}$’ is the expression of the selected gene.

Then, the patients were divided into two risk groups according to the median of risk score. The Kaplan-Meier (K-M) survival analysis was used to analyze the different prognosis between the two groups. Besides, the receiver operating characteristic (ROC) curves at 1-, 3-, and 5-years were used to study the prediction efficiency of signatures. Both signatures were further validated by the validation set. To further understand the discrimination of EMT signatures in difference stage patients, the ROC curves in four stage groups were generated and AUC values at 1-, 3-, and 5-years were calculated.

### Construction of mRNA-lncRNA Network

In order to further understand the regulatory relationship between mRNA and lncRNA, we used the Pearson test to study the interaction between mRNA and lncRNA. When the correlation coefficient |r| > 0.4 and *p* < 0.05, it is considered a significant correlation. Cytoscape (V3.7.2) was used to visualize the regulatory networks.

### Evaluation of Correlation With Immune Tumor Microenvironment

To further understand the correlation between EMT-related lncRNAs and immune features in CRC patients, the cluster of CRC cohort was performed by the unsupervised consensus approach by the “Consensus Cluster Plus” package. Because the overlapping lncRNAs between OS- and DFS-related differentially expressed EMT-related lncRNAs are the most highly conserved, we suggested they are the most likely to be associated with the progression of CRC. Therefore, the unsupervised consensus cluster analysis was performed based on the overlapping differentially expressed EMT-related lncRNAs. Meanwhile, the ESTIMATE algorithm was performed to quantify the tumor microenvironment, including immune score, stromal score, and ESTIMATE score (Yoshihara et al., 2013). The Single Sample Gene Set Enrichment Analysis (ssGSEA) algorithm was performed to quantify the fraction of 22 types of immune cells. The associations between clusters and prognosis, clinicopathological variables, and immune cells were also analyzed.

### Exploration of a Key Epithelial-Mesenchymal Transition-Related lncRNA in Colorectal Cancer

In our research, thirteen differentially expressed EMT-related lncRNAs were incorporated into the final signatures, including six to be incorporated into the OS signature and ten to be incorporated into the DFS model. Among them, there were three differentially expressed EMT-related lncRNAs, and they were included in both OS signature and DFS signature. Then, the survival curve of this three lncRNA was plotted and the log-rank test was performed. The lncRNA which was most relevant to the prognosis of CRC patients was selected for further exploration.

Gene set variation analysis (GSVA) is commonly used to estimate changes in the path and biological process activity in samples of expression datasets (Hänzelmann et al., 2013). To perform GSVA analysis, the gene sets of “c2.cp.kegg.v7.1.-symbols” were obtained from the MSigDB database. Then, we used univariate Cox regression to identify pathways related to prognosis. Pearson correlation analysis was applied to study its relationship with the immune microenvironment and prognosis-related pathways.

## Results

### Overview of Differentially Expressed Epithelial-Mesenchymal Transition-Related lncRNA

Based on the criteria, 616 CRC and 51 adjacent normal samples were included in our research. A total of 533 CRC patients with complete follow-up information, including OS and DFS, were incorporated into the survival analyses. The characteristics and clinical data of these patients are shown in Table 1. As shown in the table, there were more patients in stage I-II and most CRC patients without distant metastases.

**TABLE 1:** Clinicopathologic characteristics of CRC patients.

Characteristics	Whole cohort (*n* = 533)
Gender	
Male	289
Female	244
Age	
< 65	230
≥ 65	303
Location	
Colon	389
Rectum	144
T stage	
T1-2	113
T3-4	419
Unknown	1
N stage	
N0	316
N1-2	215
Unknown	2
M stage	
M0	408
M1	62
Unknown	63
AJCC	
I-II	301
III-IV	217
Unknown	15

**FIGURE 1:**
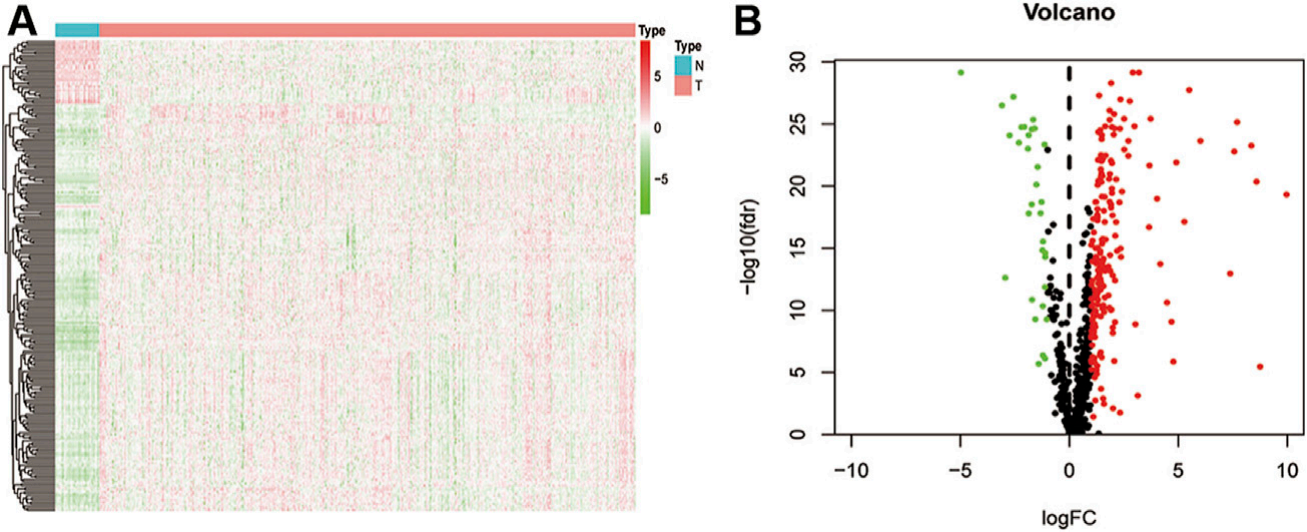
Difference analysis. **(A)** a heat map shows all differentially expressed EMT-related lncRNA between normal and CRC tissues; **(B)** a volcano map shows different high-and low-expressed EMT-related lncRNA in normal and CRC tissues.

By intersecting the EMT gene database and the TCGA database, we obtained the expression of 1,000 EMT-related mRNAs in CRC cohort. Then, 650 lncRNAs were determined as EMT-related mRNAs. To identify the CRC-specific EMT-related lncRNAs, differences between 616 primary CRC tissues and 51 adjacent normal tissues were compared to identify the differentially expressed EMT-related lncRNAs. Totally, 231 EMT-related lncRNAs were determined, which include 32 downregulated and 199 upregulated in tumors (Figure 1A and Supplementary Table S1). The volcano map also showed that the majority of EMT-related lncRNAs were upregulated in tumor tissues, indicating that these lncRNAs contribute to the tumorigenesis of CRC (Figure 1B).

### Establishment and Validation of Prognostic Signatures

Firstly, a total of 533 CRC patients with complete follow-up information were randomly divided into training and validation sets. A total of 376 patients were incorporated into the training set and 157 patients were incorporated into the validation set. The univariate Cox proportional hazard model was conducted to determine the prognostic EMT-related lncRNAs in the training set. A total of 17 and 34 EMT-related lncRNAs were identified as OS- and DFS-related EMT-related lncRNAs, respectively (Supplementary Tables S2, S3). Subsequently, stepwise model selection using the Alkaike information criterion (AIC) and multivariate Cox regression model was performed to construct two signatures, and six OS-related EMT-related lncRNAs and ten DFS-related EMT-related lncRNAs were incorporated into the final signatures (Tables 2 **and** 3). Risk scores were calculated based on the selected EMT-related lncRNAs in the final signatures (risk score of OS signature = AC127024.4*0.027 + AL591178.1*0.333 + FENDRR*-0.411 + LINC01315*-0.234 + LINC01503*0.359 + MMP25-AS1*0.543; risk score of DFS signature = AC021218.1*-0.066 + AC074117.1*-0.208 + AC104958.2*0.062 + AL031985.3*-0.905 + AL033519.3*-0.351 + AL591178.1*0.301 + BLACAT1*0.116 + LINC01503*0.245 + MMP25-AS1*0.572 + AC005837.3*0.307).

**TABLE 2:** OS-related EMT-related lncRNA for CRC patients.

lncRNA	Coef	HR	95% CI	*p*-value
AL591178.1	0.333	1.395	1.157–1.682	0.000479
LINC01503	0.359	1.432	1.169–1.754	0.00052
MMP25-AS1	0.543	1.722	1.235–2.340	0.001339
AC127024.4	0.027	1.027	1.009–1.045	0.002823
LINC01315	−0.234	0.791	0.669–0.936	0.006149
FENDRR	−0.411	0.663	0.434–1.012	0.056918

**TABLE 3:** DFS-related EMT-related lncRNA for CRC patients.

lncRNA	Coef	HR	95% CI	*p*-value
AL591178.1	0.301	1.351	1.166–1.565	6.26E–05
MMP25-AS1	0.572	1.772	1.333–2.356	8.27E–05
AL033519.3	−0.351	0.704	0.591–0.839	8.61E–05
AL031985.3	−0.905	0.404	0.251–0.652	0.0002
AC021218.1	−0.066	0.936	0.895–0.980	0.004304
AC104958.2	0.062	1.064	1.017–1.114	0.007646
AC005837.3	0.307	1.359	1.077–1.716	0.009871
LINC01503	0.245	1.278	1.037–1.575	0.021535
BLACAT1	0.116	1.124	1.004–1.258	0.043102
AC074117.1	−0.208	0.812	0.637–1.036	0.093718

**FIGURE 2:**
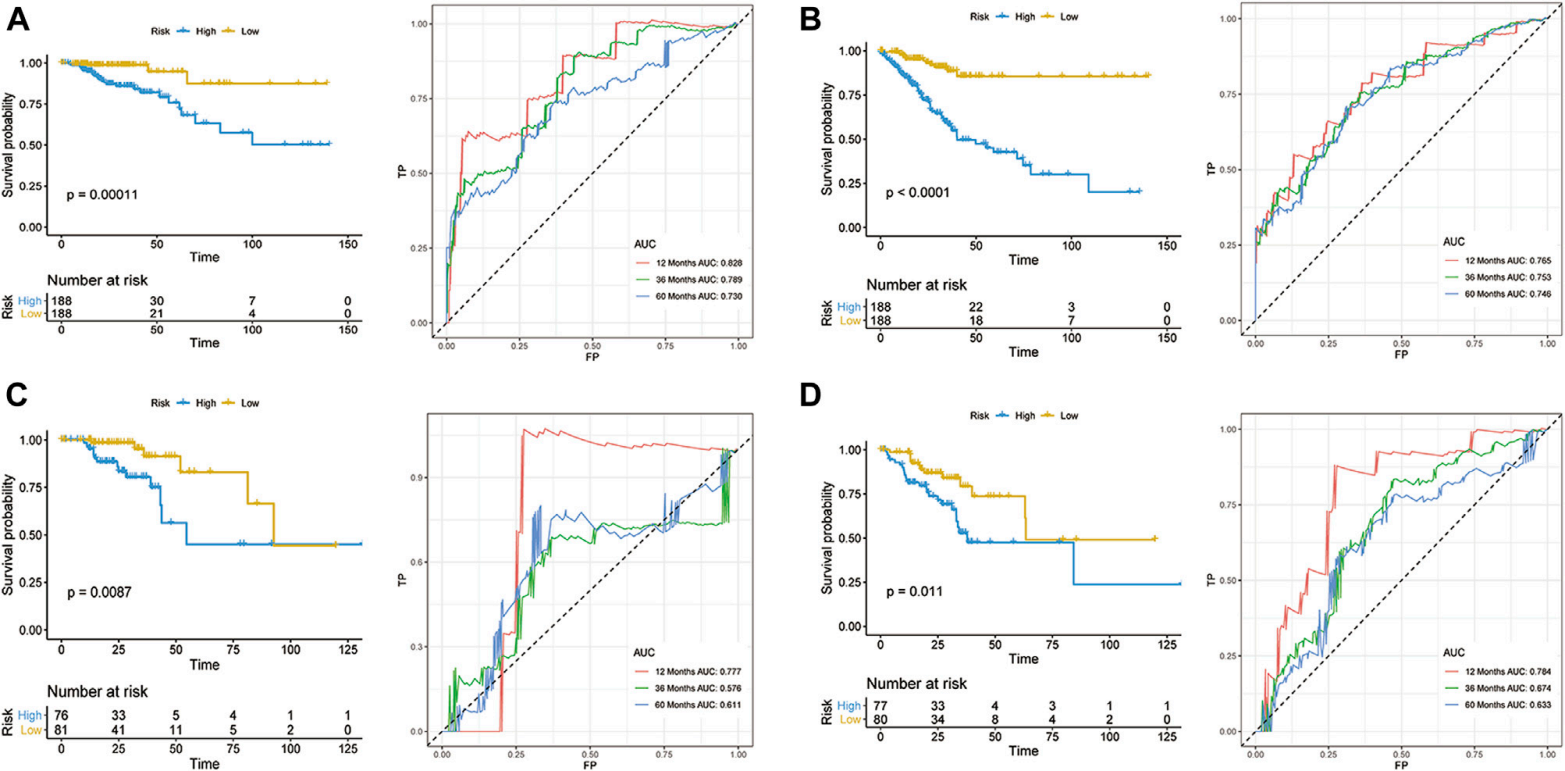
Development of prognostic models for CRC. OS **(A,C)** and DFS **(B,D)** in the training set **(A,B)** and validation set **(C,D)**.

According to the median risk score, 188 and 188 patients were grouped in the low- and high-risk groups, respectively (Figures 2A,B). The K-M survival curves and the log-rank tests showed that the OS and DFS of high-risk patients are significantly worse than that in the low-risk group. The AUC values of OS signature at 1-, 3-, and 5-years were 0.828, 0.789, and 0.730, respectively, while the corresponding AUC values of DFS signature were 0.765, 0.753, and 0.746. The above AUC values suggested the favorable discrimination of both signatures (Figures 2A,B). In the validation set, the K-M survival curves showed that the prognosis of the high-risk group is also significantly worse than that of the low-risk group and that all AUC values were higher than 0.550, indicating the stability of both signatures (Figures 2C,D).

To further understand the discrimination of signatures in subgroups, the ROC curves of both OS and DFS signatures for four stage patients are shown in Supplementary Figure S1. Overall, the discrimination of both signatures showed favorable performance in most subgroups. However, in stage I patients, the performance of both signatures showed unsatisfactory and the AUC values range from none to 0.873, which can be attributed to the better prognosis in stage I patients and stage I patients have a lower mortality and recurrence rate.

### Two mRNA-lncRNA Networks

**FIGURE 3:**
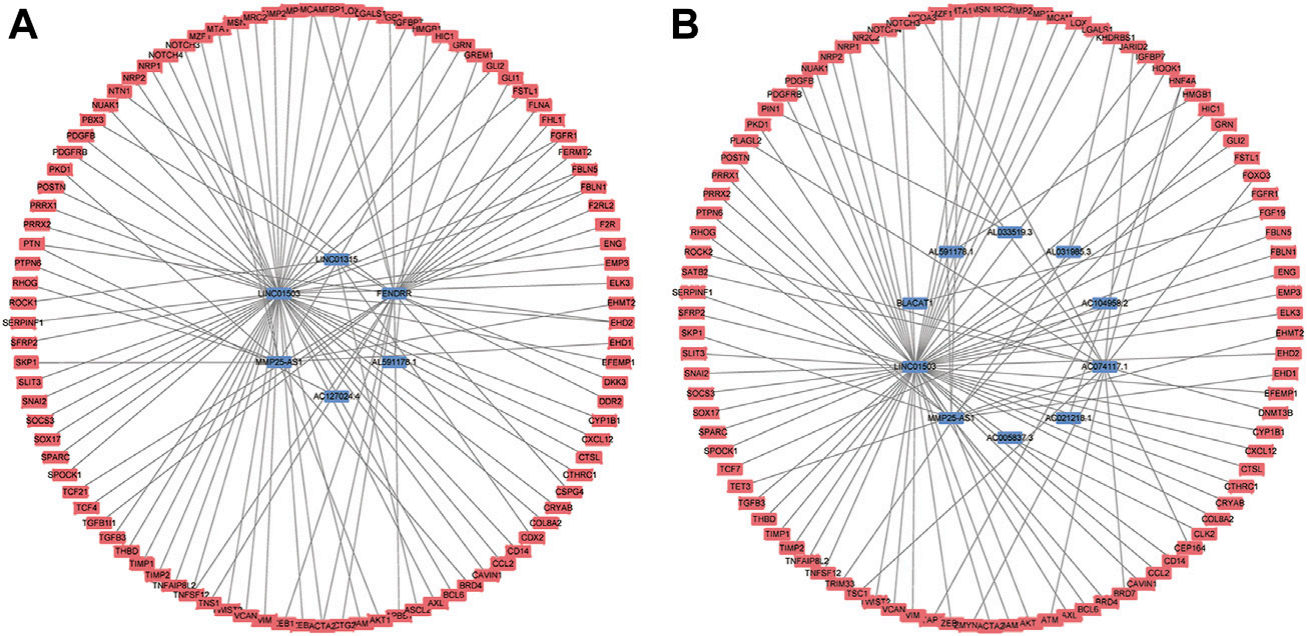
The mRNA-lncRNA co-expression network based on the correlation analysis. **(A)** the final OS signature; **(B)** the final DFS signature.

To further detect the regulatory network between mRNA and lncRNA, we then constructed the mRNA-lncRNA co-expression network based on the correlation analysis. Six lncRNAs incorporated into the final OS signature and 97 mRNAs containing 105 relationships were obtained to generate a network (Figure 3A). Meanwhile, ten lncRNAs incorporated into the final DFS signature, and 95 mRNAs containing 96 relationships were selected to generate another network (Figure 3B). The in-depth analyses of three lncRNAs that were simultaneously incorporated into OS and DFS signatures revealed that HMGB1 was predicted as the target of AL591178.1, which laid a certain foundation for further research. For the other two EMT-related lncRNAs, 11 and 60 mRNAs were confirmed as the targets of MMP25-AS1 and LINC01503, respectively.

### The Features of Gene Set Variation Analysis and Immune Cell Level

**FIGURE 4:**
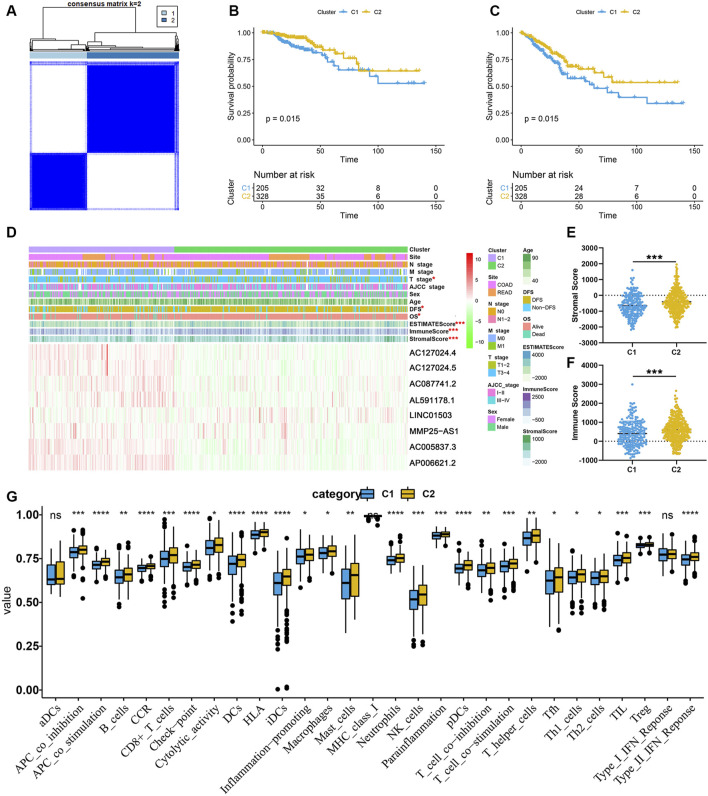
EMT-related lncRNA clusters significantly associated with the immune microenvironment. **(A)** Consensus clustering analysis identification of two clusters; **(B,C)** Kaplan–Meier survival curve of OS **(B)** and DFS **(C)** between C1 and C2; **(D)** Heat map of the lncRNA ordered by cluster, with annotations associated with each cluster; **(E,F)** Immune score and stromal score between the two clusters; **(G)** Statistical differences in each type of immune cell between C1 and C2.

To initially understand the potential mechanisms involved in the biological function of EMT-related lncRNA, the unsupervised clustering analysis was performed. According to the consensus matrix heatmap (Figure 4A), two clusters were identified (C1 (*n* = 205, 38.46%), C2 (*n* = 328, 61.54%)). The results of K-M analyses showed that the prognosis was significantly different between C1 and C2, including OS (Figure 4B) and DFS (Figure 4C).

In order to clarify the clinicopathological and immune characteristics of EMT-related lncRNA clusters, we firstly explored the relationship between clusters and clinical and immune status, which is shown in a heatmap (Figure 4D). The distribution of T stage in CRC patients between the two clusters was not random, and there were eight prognosis-related lncRNAs expressed differently between C1 and C2 (Figure 4D). Among these lncRNAs, only LINC01503 was highly expressed in C2, while the others were opposite. Besides, the ESTIMATE score, immune score and stromal score were significantly different between C1 and C2. As shown in Figures 4E,F, the stromal score and immune score of C2 are significantly higher than that of C1. At the same time, by comparing the composition of 22 immune cells, we found that the level of most immune cells is significantly higher in C2, especially immune activated cells, including CD8^+^ T cells, natural killer (NK) cells and dendritic cells (DCs) (Figure 4G). The above results indicated that the reason why C2 had a better prognosis than C1 may be related to the expression of different lncRNAs and immune mechanisms. In a word, C2 patients with favorable prognosis showed immune activation but C1 patients with unfavorable prognosis showed immune inactivation relative to immunosuppressive status.

### AL591178.1 is a Key Epithelial-Mesenchymal Transition-Related lncRNA in Colorectal Cancer

**FIGURE 5:**
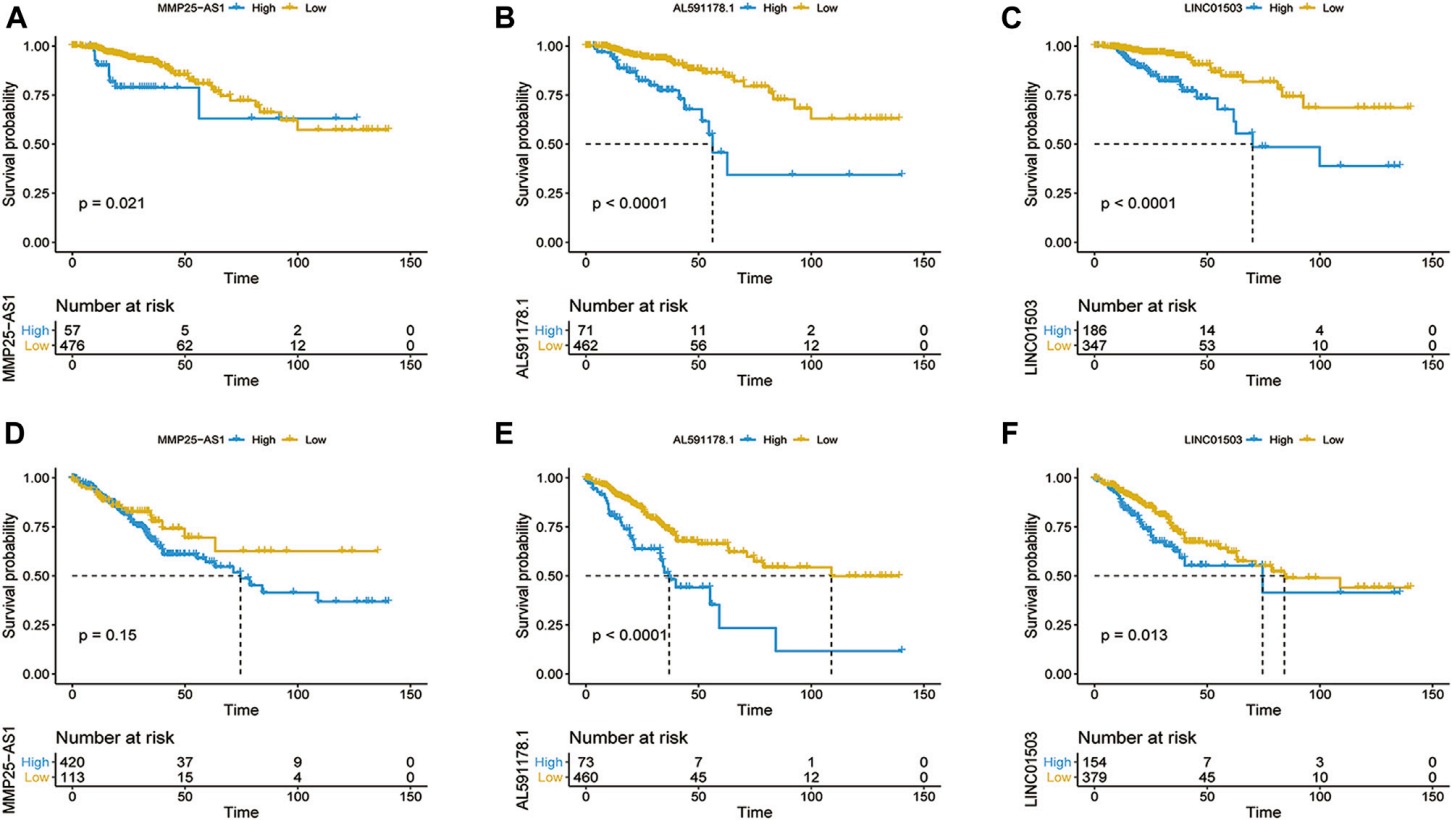
Kaplan–Meier survival curve of OS **(A–C)** and DFS **(D–F)** based on the expression of MMP25-AS1 **(A,D)**, AL591178.1 **(B,E)**, and LINC01503 **(C,F)**.

**FIGURE 6:**
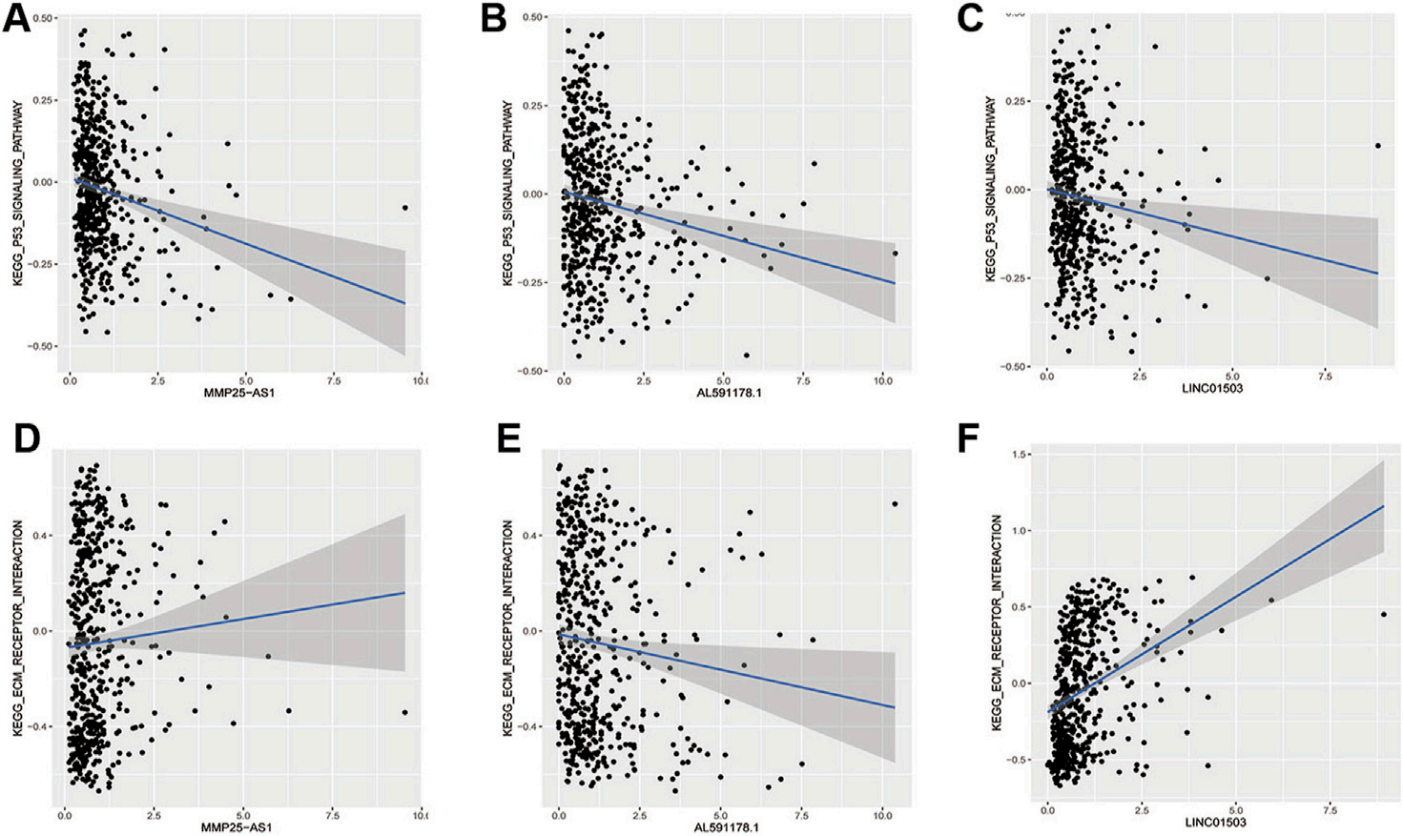
The relationship of EMT-related lncRNAs and pathways. **(A,B)** MMP25AS1 with the P53 pathway and the ECM receptor pathway; **(C,D)** AL591178.1 with the P53 pathway and the ECM receptor pathway; **(E,F)** LINC01503 with the P53 pathway and the ECM receptor pathway.

In order to find out the biomarker for CRC more accurately, we focused on three EMT-related lncRNAs that were simultaneously incorporated into OS and DFS signatures, including MMP25-AS1, AL591178.1, and LINC01503. The results showed that high expression of AL591178.1 and LINC01503 was associated with poor OS and DFS, but the differences for MMP25-AS1 were not associated with a significant difference (*p* = 0.15) (Figures 5A–F). To further explore the biological function of the three lncRNAs, the univariate Cox analysis of GSVA score was performed. A total of 21 and 48 KEGG pathways were associated with OS and DFS, respectively (Supplementary Tables S4, S5). In these significant pathways, we found that 17 common pathways were related to both OS and DFS (Supplementary Table S6). Further correlation analysis indicated that there is a high degree of correlation between the above three lncRNAs and these 17 pathways. Interestingly, two well-known pathways were detected, including the P53 signaling pathway that was associated with the tumorgenesis and immune formation of CRC and the ECM-receptor interaction pathway that was significantly associated with the formation of the tumor microenvironment (Cho et al., 2020; Yin et al., 2020; Chen Y. et al., 2020; Zou et al., 2019). The correlation plots between lncRNAs and two vital pathways are shown in Figures 6A–F.

**FIGURE 7:**
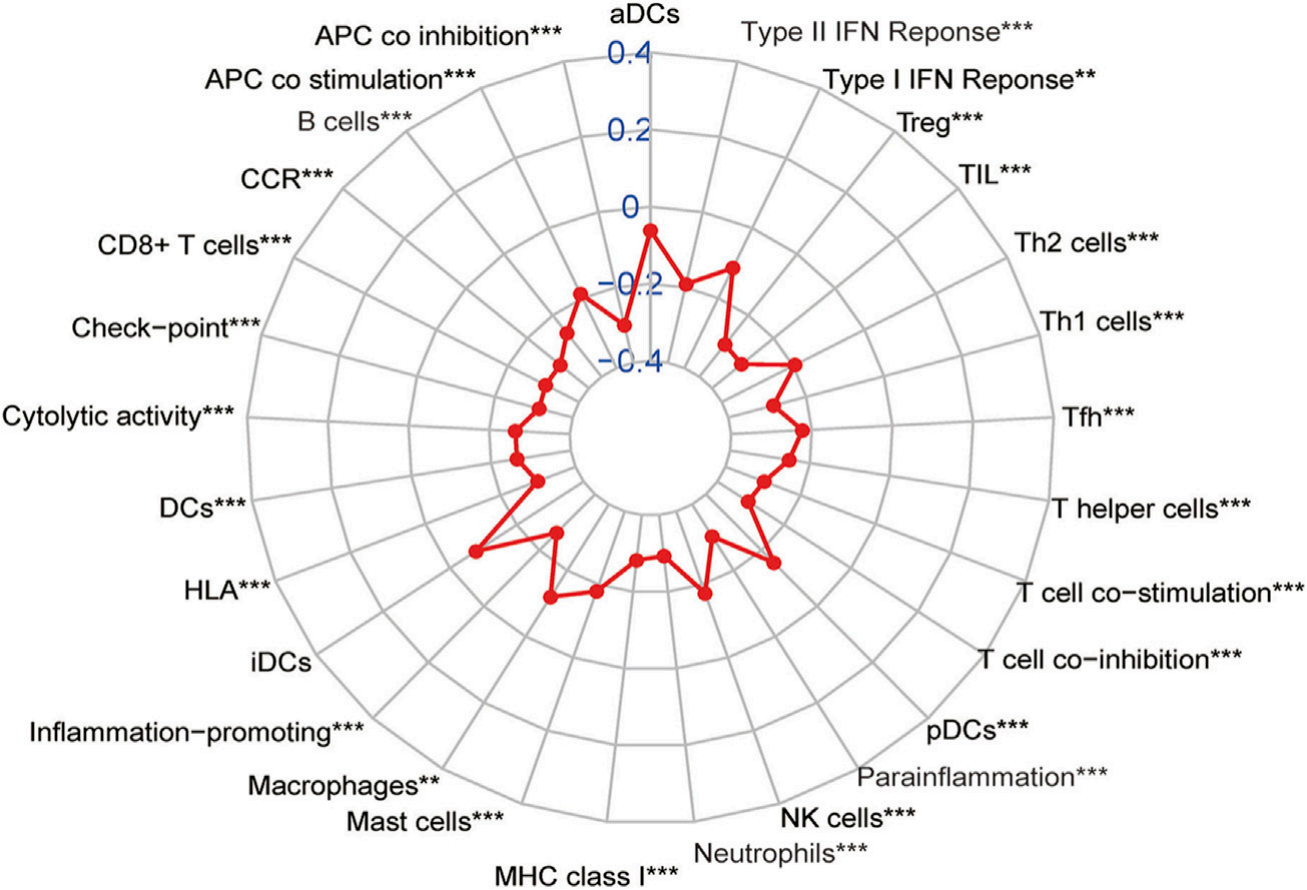
Association between 22 types of immune cells and the expression of AL591178.1.

According to previous results of our study, EMT-related lncRNA may affect the prognosis of CRC patients through immune mechanisms (Figure 4). Therefore, the lncRNA AL591178.1 was selected, which is most associated with the prognosis of CRC patients (both *p* values < 0.0001) and negatively associated with the GSVA scores of P53 signaling and ECM-receptor interaction pathways, to further study the association with the specific immune cells. As shown in the radar chart (Figure 7), the expression level is negatively correlated with the existence of immune cells, including CD4 T cells, NK cells, macrophages, and monocytes. In previous studies, we learned that the ECM-receptor interaction pathway hurts the prognosis of cancer patients (Zhang et al., 2020), but our study found that the ECM-receptor interaction pathway is negatively correlated with AL591178.1 (Figure 6D). It is speculated that AL591178.1 inhibits the tumor immune microenvironment, which may cover the prognosis of ECM’s effect.

## Discussion

Preliminary studies have shown that ERG and lncRNAs are involved in the progression of many cancers (Zou et al., 2011; Chen et al., 2013; Stavropoulou et al., 2016; Martini et al., 2017). In this study, we established two signatures to predict the OS and DFS of CRC patients, which can stratify CRC patients into significantly different risk groups to effectively predict their prognosis. Besides, the results of unsupervised consensus showed that there are higher immune score and a higher level of immune cells in C2 with a better prognosis compared with C1. In OS and DFS signatures, there are three common lncRNAs, and their expression is significantly related to prognosis. AL591178.1, as a prognostic risk factor, was found to be negatively correlated with the level of immune cells and ECM-receptor pathway. To the best of our knowledge, it is the first study to focus on the characteristics of CRC from the perspective of EMT-related lncRNAs, so as to provide a larger field of vision and choice for such patients' prediction and molecular targeting.

Normal tissues can precisely regulate the stability of genome expression and maintain their normal physiological functions. However, abnormal development of the genome is a typical feature of tumor cells, including lncRNA (Hanahan and Weinberg, 2011). In recent years, with the advancement of high-throughput sequencing technology, lncRNA, as an important genome for maintaining functions in the human body, has gradually gained attention. Our study used more common analysis methods to explore the prognostic biomarkers of CRC patients from a perspective of EMT-related lncRNA. And the results proved that our findings are accurate and reliable. For example, LINC01503 was confirmed to be correlated with the OS of CRC patients, and other studies have also found that it is highly expressed in different tumor cells, and is positively correlated with tumor cell proliferation and metastasis, including nasopharyngeal carcinoma (He et al., 2020), gastric cancer (Ding et al., 2020; Ma et al., 2020), and CRC (Wei et al., 2020). Interestingly, in cholangiocarcinoma, LINC01503 can also promote the EMT process (Qu et al., 2019), confirming that this lncRNA is an EMT-related lncRNA. Our study found that LINC01871 is a protective factor for the prognosis of CRC. He et al. (He and Wang, 2020) found that its expression level is closely related to CD8^+^ T cell enrichment levels, PD-L1 expression levels, and immune cytolytic activity, which means that LINC01871 may inhibit the growth of CRC cells by regulating the formation of immune microenvironment. Therefore, the above studies all showed the reliability of our results. Besides, some of the lncRNAs, contained in the two signatures were not reported in previous studies, such as AC127024.4 and AL033519.3, which illustrates the innovation of our study to a certain extent, and provides a theoretical basis for further research. In addition to lncRNAs, the corresponding targeting mRNA also participate immune regulation. Yang et al. (Yang et al., 2020) found that lipopolysaccharide induced the release of pro-inflammatory cytokines in a HMGB1-dependent manner to improve colon cancer progression. The discovery proved the immune role of the lncRNA and mRNA we identified.

In our results, there are differences in the levels of immune cells in C1 and C2, indicating that EMT-related lncRNA may affect the prognosis of patients through immune mechanisms. Previous studies have also confirmed it (Wang et al., 2019; Chen Q.-H. et al., 2020). The expression of lncRNA SNHG14 is up-regulated in diffuse large B cells, and its deletion will hinder the proliferation, migration, and EMT (Zhao et al., 2019). Mechanistically, SNHG14 up-regulates zinc finger E-box binding homeobox 1 (ZEB1), thereby activating PD-L1 to promote immune evasion of diffuse large B cells (Zhao et al., 2019). In order to further clarify the role of EMT-related lncRNA we found in CRC, we took the intersection of the two established signatures and found three lncRNAs related to both OS and DFS. Among them, we further focused on AL591178.1, which was negatively correlated with the infiltration level of most immune cells. The tumor immune microenvironment is an important regulatory factor of tumor development, such as CD4 T cells, CD8 T cells, and NK cells. Previous studies have found that they all have a positive impact on the prognosis of cancers (Ahlén Bergman et al., 2018; Wang et al., 2018; Wang D. et al., 2020). Therefore, we can speculate that AL591178.1 may affect the prognosis of CRC patients by regulating the abundance of immune cells. According to the GSVA function analysis and survival correlation analysis, we found that the two pathways are significantly related to the expression of AL591178.1. The P53 pathway plays a role in inhibiting EMT and invasion of cancer and is beneficial to the prognosis of patients (Wu et al., 2019; Jackson-Weaver et al., 2020), so the results of its negative correlation with the expression of AL591178.1 are consistent with their conclusion. Interestingly, the ECM-receptor pathway has been considered to promote tumorigenesis in previous studies and is negatively correlated with the prognosis of cancer patients (Zhang et al., 2016; Wang J. et al., 2020), so we speculated that AL591178.1 may suppress the tumor immune microenvironment and suppress immune function. But the mechanism involved needs further research to clarify.

Although our research is somewhat innovative, some limitations require attention. First, no independent cohort of CRC patients was used to prove that the prognosis model presented here is reproducible. Second, this study is a retrospective study, which has certain biases, and the clinical data included is not rich enough. Third, although our research has found a potential way to interfere with the prognosis of CRC patients, further experiments are needed to verify and find the corresponding target.

## Conclusion

The results we obtained highlighted the prognostic value of EMT-related lncRNA in CRC patients and explored potential mechanisms and regulatory networks. In detail, AL591178.1 was found to be a risk factor for the prognosis of CRC patients, which is inversely proportional to the level of immune cell infiltration, P53 pathway, and ECM-receptor pathway. Thus, this study identified a novel potential target to provide treatment opportunities for CRC patients.

## Data Availability

The original contributions presented in the study are included in the article/Supplementary Material, further inquiries can be directed to the corresponding authors.
