# Supplementary figures and images for: Identification of Epithelial-Mesenchymal Transition-Related lncRNAs that Associated With the Prognosis and Immune Microenvironment in Colorectal Cancer
Source: Front Mol Biosci. 2021 Apr 1;8:633951. doi: 10.3389/fmolb.2021.633951 (PMC8059639; doi:10.3389/fmolb.2021.633951)

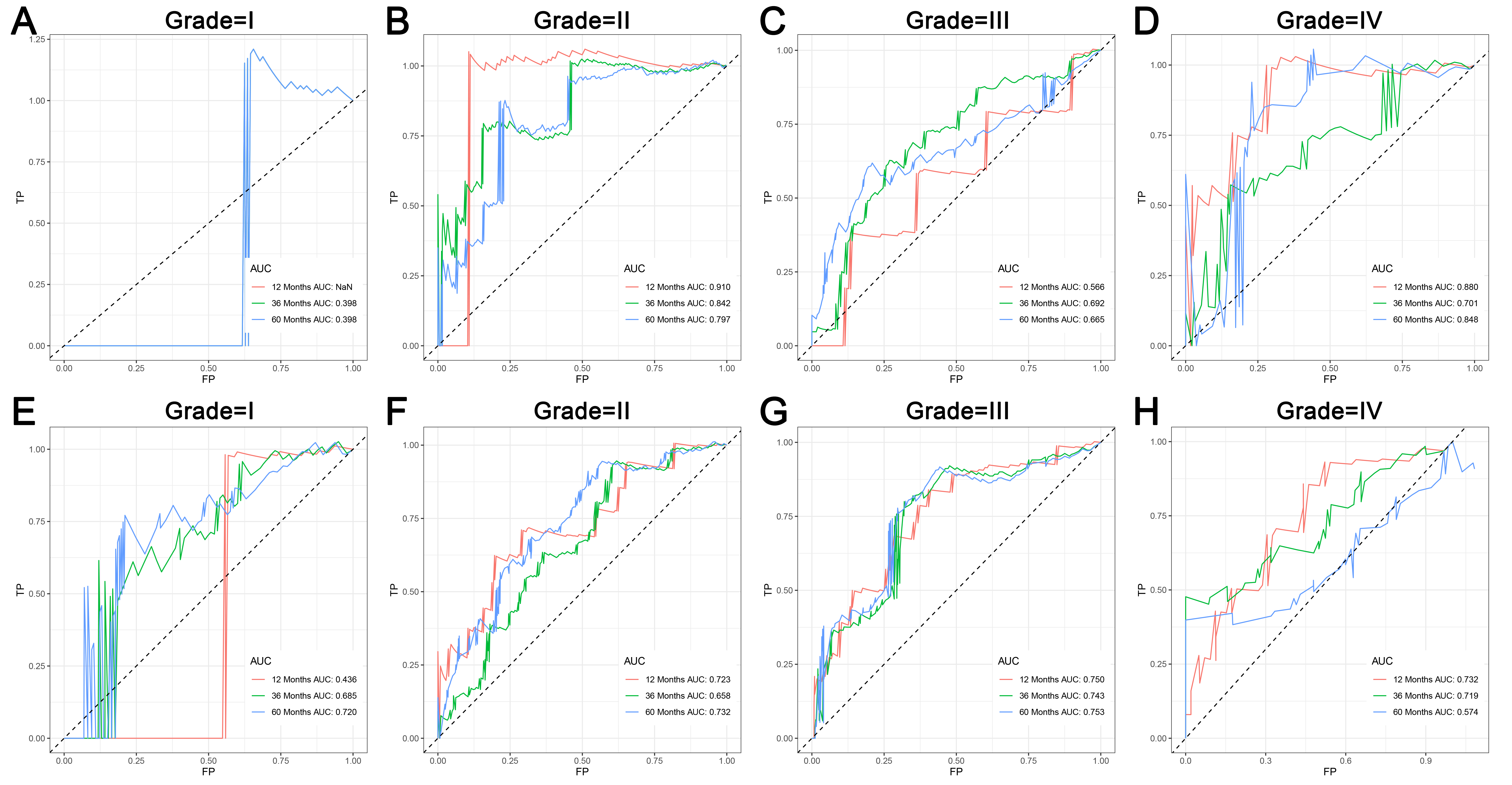

Supplement: Supplementary file 9 [file image1.tif]
